# The emerging roles of phosphatases in Hedgehog pathway

**DOI:** 10.1186/s12964-017-0191-0

**Published:** 2017-09-20

**Authors:** Long Zhao, Liguo Wang, Chunli Chi, Wenwen Lan, Ying Su

**Affiliations:** 1 Cardiovascular Research Center, Department of Medicine, Massachusetts General Hospital and Harvard Medical School, Charlestown, MA 02129 USA; 2 Institute of Evolution & Marine Biodiversity, College of Marine Life Sciences, Ocean University of China, Qingdao, 266003 China

**Keywords:** Hedgehog pathway, Phosphorylation, Phosphatase, Kinase

## Abstract

Hedgehog signaling is evolutionarily conserved and plays a pivotal role in cell fate determination, embryonic development, and tissue renewal. As aberrant Hedgehog signaling is tightly associated with a broad range of human diseases, its activities must be precisely controlled. It has been known that several core components of Hedgehog pathway undergo reversible phosphorylations mediated by protein kinases and phosphatases, which acts as an effective regulatory mechanism to modulate Hedgehog signal activities. In contrast to kinases that have been extensively studied in these phosphorylation events, phosphatases were thought to function in an unspecific manner, thus obtained much less emphasis in the past. However, in recent years, increasing evidence has implicated that phosphatases play crucial and specific roles in the context of developmental signaling, including Hedgehog signaling. In this review, we present a summary of current progress on phosphatase studies in Hedgehog pathway, emphasizing the multiple employments of protein serine/threonine phosphatases during the transduction of morphogenic Hedgehog signal in both *Drosophila* and vertebrate systems, all of which provide insights into the importance of phosphatases in the specific regulation of Hedgehog signaling.

## Background

The Hedgehog (Hh) pathway is a conservative ligand-dependent cellular signaling mechanism, playing a vital role in diverse biological processes, such as cell proliferation and differentiation, embryonic development, and maintenance of stem cell status in adults [1]. Aberrant Hh signaling activities have been implicated in many human disorders including birth defects and cancers [2, 3]. Therefore, the activity of Hh signaling is required to be precisely controlled.

Protein phosphorylation is one of the most important and well-studied post-translational modifications [4]. Nearly one-third of proteins in cells are subject to at least one-time phosphorylation during their whole lives [5]. Protein phosphorylation is a reversible process, mediated by two types of enzymes: protein kinase and protein phosphatase [6]. A protein kinase is responsible for transferring a phosphate group from ATP to a serine, threonine or tyrosine residue at a substrate protein, while a phosphatase is in charge of removing phosphates from the substrate. The balance between kinase and phosphatase activities controls phosphorylation status of a substrate protein, alteration of which is capable of affecting its almost every aspect, such as conformation/structure, stability, activity, protein-protein interaction [6]. In contrast to protein kinases, protein phosphatases have been much less studied. They were initially considered as possessing broad and constitutive activities without functional specificities. However, increasing evidence is indicating that protein phosphatases are regulated in complex manners and are highly specific towards different protein substrates [6].

In the context of developmental signal transduction, protein phosphorylation has been revealed to play a critical role in precisely controlling the status and amplitude of signaling pathways [6]. In Hh signaling pathway, several core components have been found to undergo phosphorylations, which significantly contribute to proper controls of Hh signaling outcomes [7]. Although the executing kinases in these phosphorylation events have been intensely studied [8], relatively little is known about the responsible protein phosphatases. In this review, we mainly summarize emerging studies of phosphatases involved in regulation of Hh signaling in recent years, with a highlight of multiple employments of protein phosphatase 2A (PP2A), one of abundant and important cellular protein phosphatases, during Hh signal transduction cascade, emphasizing the equal importance of phosphatase as kinase in regulating Hh signaling.

## Principles of Hh signaling transduction

**Fig. 1:**
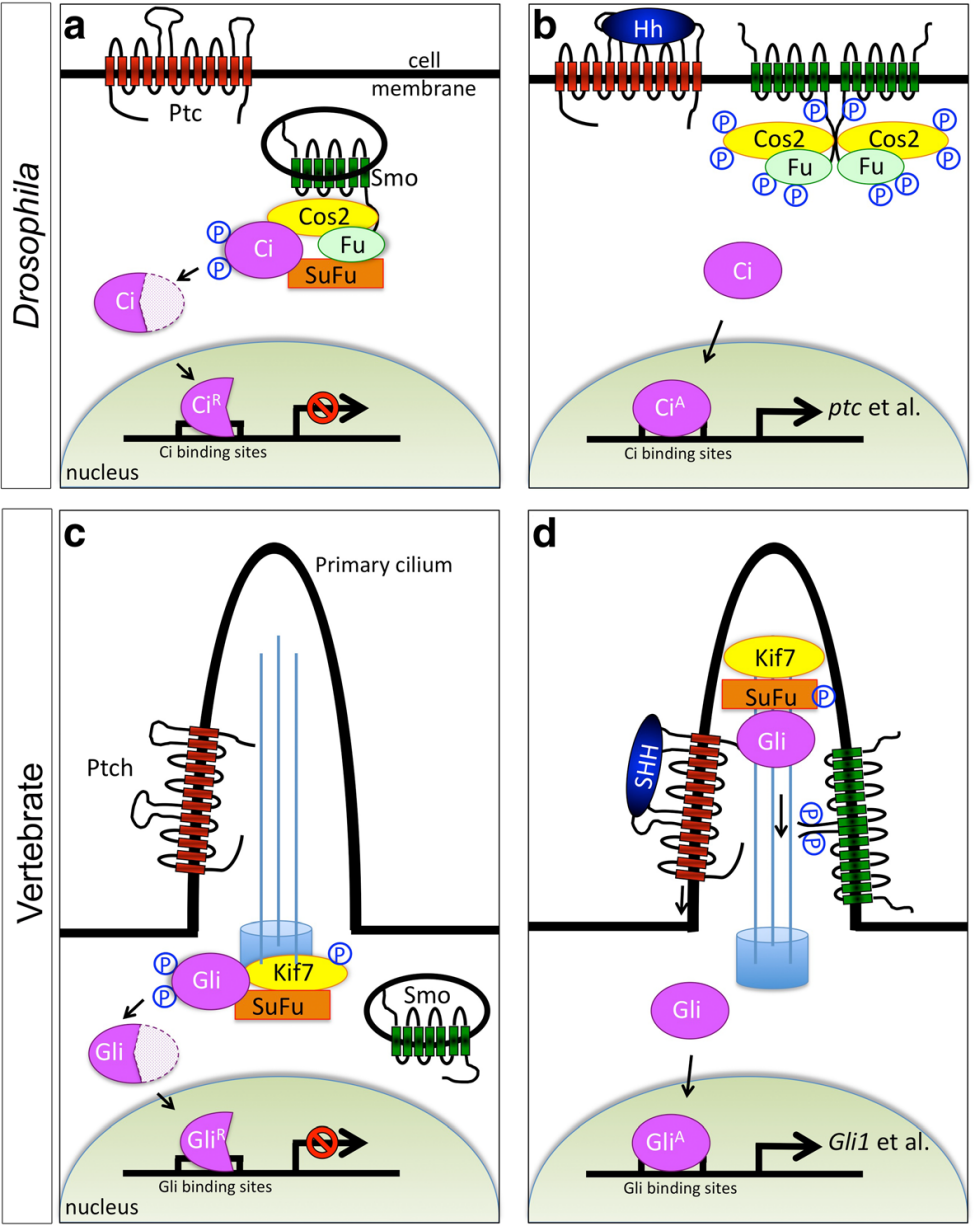
Schematic of Hh signal transduction in *Drosophila* and vertebrates. **a** and **b** In *Drosophila*, without Hh ligand, the phosphorylated transcription factor Ci undergoes proteolytic cleavage to produce a truncated form as a repressor for target gene transcription (**a**). With Hh, the pathway activator Smo is relieved from receptor Ptc inhibition to get phosphorylation and plasma membrane accumulation, which triggers a series of phosphorylation events on Cos2 and Fu. Eventually, full-length Ci is stabilized to enter nucleus activating target gene expression (**b**). **c** and **d** In vertebrates, the principal rule of Hh signal transduction is conserved. However, several differences are indeed existing: signaling activation takes place at the primary cilia instead of plasma membrane, Sufu plays a more critical role to transduce signal rather than Fu, and Cos2 homolog Kif7 obtains phosphorylation at basal condition but not during Hh signal activation

Since the original discovery of *hh* gene in *Drosophila melanogaster* as a regulator of body patterning during embryonic development, the knowledge about principle mechanism of Hh signal transduction has dramatically increased over the past decades [1, 9]. The intense genetic research in *Drosophila* has elucidated the core Hh signal transduction cascade (Fig. 1 a and b), which is initiated by two transmembrane proteins, a signal receptor Patched (Ptc) and an essential signal activator Smoothened (Smo). In the absence of Hh ligand, Ptc inhibits Smo activity, probably by preventing its cell surface localization. A transcription factor Cubitus interruptus (Ci) is proteolytically processed, which is facilitated by a cytoplasmic signal transducer complex consisting of Costal2 (Cos2), Fused (Fu), and Suppressor of Fused (Sufu), to produce a transcriptional repressor CiR for Hh target genes (Fig. 1a). Once Hh binds to Ptc, Smo is relieved from Ptc inhibition and becomes activated, eventually resulting in the stabilization of Ci, which is converted to a transcriptional activator CiA to replace CiR in nucleus and switch on the transcription of Hh target genes (Fig. 1b) [10–17].

The core Hh signal transduction shares same principle from *Drosophila* to mammals, although the mammalian pathway is more complex owing to the presence of multiple ligands, receptors and transcription factors (Table 1) [18]. This complication can be representatively reflected by the diverse functions of Glioma-associated oncogene homologue (Gli) proteins, the Ci homologous proteins in vertebrates. Three members are known in Gli family: Gli1, Gli2, and Gli3. Gli1 mainly serves as a target gene of Hh signaling. Gli2 and Gli3 share the transcriptional task of Ci in Hh signaling: Gli2 preferredly contributes to the activator form GliA, and Gli3 is the major source of repressor form GliR [19, 20].

**Table 1:** Core components of Hh pathway in *Drosophila* and vertebrates

Component Function	Protein Type	*Drosophila* Protein	Vertebrate Protein
Ligand	Secreted protein	Hedgehog (Hh)	Sonic Hedgehog (Shh), Desert Hedgehog (Dhh), Indian Hedgehog (Ihh)
Receptor	12-transmembrane protein	Patched (Ptc)	Patched1 (Ptch1), Patched2 (Ptch2)
Transcriptional activator and repressor	Zinc finger transcription factor	Cubitus interruptus (Ci)	Glioma-associated oncogene homologue (Gli1–3)
Signal activator	7-transmembrane protein, G-protein-coupled-receptor (GPCR)	Smoothened (Smo)	Smo
Signal transducer	Kinesin-like protein	Costal2 (Cos2)	Kinesin family member 7 (Kif7)
	Ser/Thr Kinase	Fused (Fu)	Fu/STK36
	PEST domain protein	Suppressor of Fused (Sufu)	Sufu

Distinct from *Drosophila* membrane-mediated Hh pathway, vertebrate Hh pathway is transduced in a manner depending on primary cilium, a microtubule-based membrane protrusion and antenna-like cellular structure, although the exact biochemical mechanisms remain largely unclear (Fig. 1 c and d) [1]. Hh-induced Smo accumulation on primary cilia and the following transportation of Gli proteins to tips of cilia are prerequisite steps for Gli nucleus translocation. As most *Drosophila* cells lack cilium structure during development, it was thought that cilia-mediated Hh signaling is restricted within vertebrates. However, intriguingly, a cilia-mediated Hh pathway in *Drosophila* olfactory sensory neurons was characterized recently [21], indicating that ciliary Hh pathway is also conserved in *Drosophila* system.

## Major phosphorylation events in Hh pathway: A kinase view

Protein phosphorylation represents one of the most common post-translational modifications in eukaryotes. Not surprisingly, it also occurs on multiple components during Hh signal transduction [7]. During the past decades, phosphorylation events in Hh pathway have been extensively studied, mainly focusing on the characterization of executing kinases [8], reflecting a fine-tuned responding mechanism for cellular components to precisely transduce Hh signal.

Smo, a seven-pass transmembrane protein with a long carboxyl-terminal intracellular tail, is one of the best-studied components for phosphorylation modification in Hh pathway. Upon Hh stimulation, Smo protein undergoes multiple phosphorylations at its intracellular tail [22], by which Smo is activated to transduce signals towards downstream effectors. In *Drosophila*, a sequential phosphorylation by cAMP-dependent protein kinase (PKA) and casein kinase I (CK1) [22–24] is the most critical step to inhibit Smo ubiquitination and its subsequent endocytosis and degradation [25, 26], resulting in Smo cell surface accumulation. Moreover, these PKA-CK1 phosphorylations drive a conformation switch of Smo cytoplasmic tail from a closed inactive to an open active form [27], facilitating Smo maximal phosphorylation by other kinases, such as G-protein-coupled receptor related kinase 2 (GRK2/Gprk2) and CKIγ/Gilgamesh(Gish) [28–32], to achieve full activation of Smo. However, these PKA-CK1 clusters are not found at vertebrate Smo. Instead, GRK2 and CK1 were thought to replace the role of PKA-CK1 in activating vertebrate Smo by promoting its ciliary localization and active conformation [28, 29, 31, 33–36].

Ci/Gli protein, the transcription effector of Hh pathway, is another key component modulated by phosphorylations, and its phosphorylation events exhibit high conservation between *Drosophila* and vertebrates. It has been well established that multiple-sites phosphorylations on Ci/Gli by PKA, PKA-primed CK1, or PKA-primed glycogen synthase kinase 3 (GSK3), when Hh signal is off, facilitate the recruitment of Cullin1-based E3 ubiquitin ligase complex containing a F-box protein Slimb (*Drosophila*) or β-TrCP (vertebrates), producing a truncated transcriptional repressor CiR/GliR through proteolytic processing [37–45]. In contrast, when Hh signal is switched on, the transcriptional activator form of Ci/Gli, converted from full-length Ci/Gli, is eventually subject to complete degradation catalyzed by another Cullin3-based E3 ubiquitination ligase complex that contains HIB/Roadkill (*Drosophila*) or SPOP (vertebrates) [46–48]. The association between Ci/Gli and HIB/SPOP can be disrupted by CK1-mediated phosphorylation at multiple serine/threonine-rich degrons on Ci/Gli, which are distinguished from those PKA-primed CK1 sites, as a consequence, protecting CiA/GliA from premature degradation [49]. Additionally, several other kinases, such as atypical protein kinase C (aPKC), casein kinase 2 (CK2), dual-specificity tyrosine phosphorylation-regulated kinases (DYRKs), were also implicated in the regulation of Ci/Gli activity [50–53].

The cytoplasmic Cos2-Fu-Sufu complex serves as a bridge between Smo and Ci/Gli to transduce Hh signaling from cell surface to nucleus [54–56]. In response to Hh signal, in *Drosophila*, Fu kinase phosphorylates Cos2 and Sufu proteins, very likely in a direct manner, to trigger the dissociation of Cos2-Fu-Sufu-Ci complex [55, 57–60], promoting Ci release from the complex and its subsequent activation [57, 61]. Fu itself is also subject to phosphorylation to obtain full activity, including autophosphorylation and its primed CK1 phosphorylation [54, 55, 57, 62, 63]. In mammals, Fu homologs have been suggested as two proteins STK36/Fu and Ulk3. However, it is unlike that they function similarly as Fu in phosphorylating Cos2 and Sufu, as mouse STK36/Fu appears to be dispensable for embryonic development [64, 65] and Ulk3 phosphorylates Gli proteins in vitro [66, 67]. Instead, an unknown kinase phosphorylates vertebrate homologous protein of Cos2, kinesin superfamily member 7 (Kif7) [68], while PKA and GSK3 control mammalian Sufu phosphorylation [69].

## The emerging study of phosphatase in Hh pathway

In contrast to kinases, the participation of phosphatase in Hh pathway and the underlying mechanistic details are poorly understood. Recently, increasing evidence is reported to imply an equally important role of the phosphatase to kinase for the modulation of Hh signaling. According to the type of targeting phosphor-residue, protein phosphatases are classified into three major groups: tyrosine phosphatase, serine/threonine phosphatase, and dual-specificity phosphatase [70]. To date, the majority of known phosphorylation events in Hh pathway are taking place at serine or threonine residues [7]. Correspondingly, protein serine/threonine phosphatases currently attract most attentions in the studies of phosphatase function during Hh signal transduction.

### Protein phosphatase 1

Protein phosphatase 1 (PP1) belongs to serine/threonine phosphatase family, and together with protein phosphatase 2A (PP2A), accounts for more than 90% of protein phosphatase activities in eukaryotes [71]. As such an abundant phosphatase, it is not surprising that PP1 can regulate Hh signal. Actually, the biochemical and genetic studies in *Drosophila* cultured cells and wing imaginal discs have systematically demonstrated that PP1 negatively modulates Hh signaling activities through specifically reverting PKA-mediated phosphorylation of Smo protein [72]. The role of PP1 as a phosphatase regulator for Hh pathway was also uncovered in a genome-wide in vivo RNA interference (RNAi) screen searching for kinases and phosphatases that regulate Wnt and/or Hh signaling pathways [73].

*Drosophila* genome encodes four PP1 catalytic subunits (PP1c) by two subtypes of genes: *PP1α* and *PP1β* [74, 75]. Three genes encoding *PP1α* isozymes are named as *Pp1-13C*, *Pp1-87B*, and *Pp1-96A*, according to their chromosomal locations. The fourth gene, *flapwing* (*flw*), codes for *PP1β* subtype. Smo was detected to interact with all four PP1cs in cultured cells, and individually knocking down these PP1cs by RNAi induced similar levels of Smo phosphorylation [72]. However, regarding specificity of each PP1c in regulating Hh signal activities, Flw seems to act as a positive regulator of Hh pathway, whereas three of PP1α isozymes were observed to negatively modulate Hh signaling outcomes represented by Hh-responsive gene expressions [73]. Even though the mechanism underlying these distinguished effects is not clear, these functional differences between PP1α and PP1β/Flw have been found in other contexts. For examples, Flw, but not PP1α, binds to *Drosophila* myosin phosphatase targeting subunit MYPT-75D, functioning as a non-muscle myosin phosphatase to dephosphorylate the nonmuscle myosin regulatory light chain Spaghetti Squash (Sqh) [76]. Furthermore, PP1α does not rescue semi-lethality of *flw* mutants, and Flw also does not rescue *PP1α* double mutants, suggesting non-redundant functions of PP1α and PP1β/Flw during development [77].

### Protein phosphatase 2A

PP2A is a highly and broadly expressed phosphatase in eukaryotes with the involvements in a wide range of biological processes [6]. In special, PP2A was thought to act as a tumor suppressor, which was initially indicated by the discovery of its inhibitor okadaic acid as a potent tumor promoter, later supported by the finding of its interaction with oncoproteins [78, 79]. Thus far, PP2A is the most frequently studied seine/threonine phosphatase in Hh pathway. PP2A was initially linked to Hh signaling in mammalian cultured cells [80]. In this study, inhibiting PP2A activity by okadaic acid treatment blocks the expression of COUP-TFII, a Gli-independent Shh responsive target. Consistently, PP2A catalytic subunit overexpression mimics Shh stimulation to induce this target expression. In *Drosophila*, PP2A also appears to be required in Hh pathway. *Microtubule star* (*mts*), which encodes the unique PP2A catalytic subunit in flies, was identified as a gene required for maximal Hh signaling activation from an in vitro RNAi screen in cl-8 cultured cells [81]. In this study, knocking-down *mts* resulted in the reduction of Hh-induced reporter activity. Consistently, in a deficiency screen for genomic regions that enhance or suppress a *smo* partial loss-of-function wing vein phenotype, *mts* was found to positively regulate Hh signaling due to the observation that *mts* loss-of-function mildly enhanced the *smo* knock-down (RNAi) phenotype [82]. In a more recent genome-wide in vivo RNAi screen for the phosphor-regulators of multiple signaling pathways, the involvement of PP2A in Hh signaling pathway was indicated again [73].

The clue for clarifying PP2A targets in Hh pathway has been obtained from a *mts* overexpression study [81], in which overexpressing *mts* doubled Hh-responsive reporter activity in Hh-uninduced cells but reduced the reporter activity in half in Hh-stimulated cells. Interestingly, PKA showed similar effects on Hh reporter activity as that of *mts*, suggesting a possibility that PKA and PP2A act on similar substrates. As described above, both Smo and Ci have been characterized as the substrates of PKA [7]. PP2A may similarly modulate both Smo and Ci dephosphorylations. Indeed, more intensive studies have demonstrated that PP2A plays multiple roles in dictating signaling output by regulating Smo, Ci/Gli, and even Cos2/Kif7 [72, 83–86].

#### PP2A regulates Ci/Gli with elusive molecular mechanisms

As the transcription factor of Hh pathway, Ci/Gli activity is extremely critical for Hh signaling outcomes. PP2A has been implicated to affect almost every steps of Ci/Gli activation, including Ci/Gli protein phosphorylation, proteolytic processing, nuclear localization, transcriptional activity and degradation, in an either direct or indirect manner. In *Drosophila*, PP2A promotes Ci dephosphorylation and attenuates Ci cleavage, therefore, positively regulating Hh signaling outputs [83], which is consistent with the results from previous screen studies [81, 82]. In vertebrates, PP2A likely regulates Gli in a different way. In a variety of mammalian cancer cell lines with self-activated Shh signaling, increasing PP2A activity led to cytosolic retention of full-length Gli3 and its decreased transcription activity, while inhibition of PP2A enhanced Gli3 nuclear accumulation and its transcriptional activity [85, 86]. This negative regulation of PP2A in Gli3 transcriptional activity conflicts with the knowledge that Gli3 undergoes phosphorylation-dependent cleavage to produce a transcriptional repressor of target genes, strongly arguing against the direct mode of PP2A regulation in Gli3 localization and activity, and suggesting a possible involvement of other PP2A-modified factors in Hh pathway. As supporting evidence, PP2A was found to indirectly down-regulate the stability of Gli proteins by controlling the dephosphorylation of Dzip1 [87], a ciliogenesis regulator known in zebrafish [88].

#### PP2A dephosphorylates Smo as a checkpoint factor to restrict Hh-induced tissue overgrowth

In addition to Ci/Gli, PP2A also modulates Smo phosphorylation and activity. As known, Smo is subject to sequential phosphorylations mediated by PKA and then CK1 in *Drosophila* in response to graded Hh stimulation [7]. This CK1-mediated hyperphosphorylation of Smo requires a high threshold of Hh signal, promotes Smo trafficking to plasma membrane, and confers Smo maximal activity to activate downstream signal transduction. PP2A was demonstrated to specifically counteract with CK1 to dephosphorylate Smo, consequently, blocking Hh-induced Smo membrane accumulation and target gene expressions [72]. Theoretically, PP2A is capable of serving as a checkpoint factor to restrict the inappropriate signal activities induced by overdosed Hh signal. However, this PP2A action on Smo dephosphorylation in *Drosophila* might not be conserved in vertebrate system, because these PKA-primed CK1 consensus sites are not found on vertebrate Smo. Consistently, okadaic acid treatment of MEFs was not able to alter the Hh-dependent localization of Smo in cilia [84]. Instead, another Hh pathway component Cos2/Kif7 was discovered as a direct PP2A substrate in vertebrates.

#### PP2A dephosphorylates Kif7 as a positive effector on vertebrate Hh signaling

PP2A inhibitor okadaic acid inhibits Kif7 trafficking in cilia and blocks Hh signaling [84]. Conserved with Cos2, the phosphorylation of Kif7 directs its subcellular localization and the transcriptional output of Hh pathway. However, unlike Cos2, Kif7 is phosphorylated under basal conditions and is dephosphorylated in response to Hh signaling [84]. Mass-spectrum analysis has identified three phosphorylation sites on mouse Kif7, of which Ser1337 is a most critical site for Kif7 cilia localization and Hh signaling activation [84]. Indeed, PP2A exactly dephosphorylates this residue of Ser1337 at mouse Kif7 in the presence of Hh signal, triggering Kif7 localization to the tips of primary cilia and inducing the Gli-mediated transcriptional output of Hh signaling [84]. However, it remains unclear whether PP2A regulates Cos2 in fly. In addition, the kinase responsible for Kif7 phosphorylation remains to be uncovered. Although Cos2 phosphorylation is Fu-dependent in *Drosophila*, mouse Fu appears to play no role in Kif7 phosphorylation [60, 64].

#### PP2A substrate selection is controlled by its distinct regulatory subunits

**Fig. 2:**
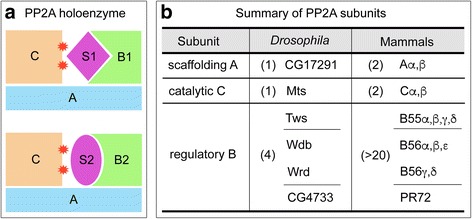
Subunit composition of PP2A holoenzyme. **a** PP2A holoenzyme is a heterotrimeric complex, consisting of three subunits: a scaffolding subunit A, a regulatory subunit B, and a catalytic subunit C, in which B subunit confers the specificity of substrates, by providing distinguished docking surfaces (represented by B1 and B2) towards the active sites (indicated by red asterisks) of C subunit, to allow the binding of specific substrate proteins (S1 and S2, respectively). **b** *Drosophila* genome encodes a single A subunit and a single C subunit, but four B subunits. In mammals, there are two A subunits, two C subunits, and more than 20 B subunits. The B subunits are classified into at least three families: B55, B56, and PR72, of which B56 family includes the largest number of members, and is further divided into two subgroups: Wdb/α,β,ε and Wrd/γ,δ. The diversity of B subunit contributes to the selection of various PP2A substrates. Tws, Twins; Wdb, Widerborst; Wrd, Well-rounded

The existence of multiple PP2A targets in Hh pathway raised an important question of how PP2A selects its substrate. The character of PP2A functioning as a heterotrimeric complex might be the key to answer this question. Protein phosphatase counteracts kinase to modulate the phosphorylation status of substrate protein. In mammals, there are around 400 serine/threonine kinases, but intriguingly, there are only about 40 protein serine/threonine phosphatases [71, 89]. Such efficient employment of protein serine/threonine phosphatases in counteracting kinases is achieved by forming numerous multimeric holoenzymes with other interacting partners, each with its own substrates and mode of regulation. This concept of holoenzyme has been well illustrated for PP2A [71]. PP2A holoenzyme is a heterotrimeric complex, composed of a scaffolding subunit, a regulatory subunit and a catalytic subunit, of which the number of regulatory subunits is much higher than that of scaffolding or catalytic subunit (Fig. 2 a and b) [90]. Through the combinatorial association of multiple subunits, and with the existence of alternative splicing, PP2A achieves a large diversity of holoenzyme composition [91]. The crystal structure analysis of PP2A heterotrimeric holoenzyme has revealed that highly acidic concave side of regulatory subunit, towards the active sites of catalytic subunit, outlines a docking pocket to recruit substrate proteins [92]. Different regulatory subunit possesses distinguished charged concave surface, allowing distinct substrate proteins fitting into the docking region (Fig. 2a). Therefore, the regulatory subunit of a PP2A trimeric complex confers the substrate specificity of PP2A holoenzyme.

As PP2A seems to target more than one component in Hh pathway, thereby, the roles of PP2A regulatory subunits in recognizing distinct substrates in Hh pathway were explored. In mammals, three major PP2A regulatory subunit families, B/B55, B′/B56, and B″/PR72, have been classified (Fig. 2b), of which B56 family comprises the largest and most conserved regulatory subunit family [93]. Five B56 family members have been identified in mammals, including α, β, γ, δ, and ε. Depletion of B56ε in *Xenopus* embryos reduced the Shh-induced target gene *ptc-1* expression, indicating that B56ε is required for Hh signaling activity [94]. More analysis showed that Hh pathway upstream of Gli remains intact in B56ε-depleted embryos, further demonstrating that B56ε likely regulates Hh pathway at the level of Gli during *Xenopus* development [94].

In *Drosophila*, four regulatory subunits are encoded: Twins (Tws) represents B55 family, Widerborst (Wdb) and Well-rounded (Wrd) belong to B56 family, and CG4733 is the member of PR72 family. According to the evolutional analysis, B56 family members can be further divided into two clades: Wdb/B56αβε and Wrd/B56γδ [91]. In consistent with B56ε functions in *Xenopus*, Wdb was first identified as a phosphor-regulator of Hh pathway from a genome-wide RNAi screen [81], then Wdb-containing PP2A was found to prevent Ci phosphorylation and proteolytic processing [83]. However, Wdb failed to be immunoprecipitated with Ci and did not affect Ci cellular localization [72]. Instead, Tws from B55 family, which has previously been associated with Wnt/Wingless signaling [95], is able to interact with Ci and promote Ci nuclear localization [72], suggesting that Tws and Wdb may play distinct roles to modulate Ci phosphorylation and localization. In terms of Smo dephosphorylation, Wdb-containing PP2A holoenzyme counteracts with CK1 to control Smo hyperphosphorylation status, by which Wdb negatively modulates Smo cell surface accumulation and Hh signaling activities [72]. And this PP2A regulation on Smo is specifically controlled by Wdb, as manipulating Tws, Wrd, or CG4733 expression failed to alter Smo cellular localization and phosphorylation [72]. Therefore, PP2A regulatory subunits exhibit specific preferences in the formation of distinct PP2A holoenzyme to dephosphorylate Smo or Ci in *Drosophila*.

### Protein phosphatase 4

In addition to PP2A, another known phosphatase regulating Smo is protein phosphatase 4 (PP4). Knocking down *pp4* by RNAi was able to promote Smo phosphorylation, but failed to induce Smo cell surface accumulation [83], suggesting that Smo phosphorylation status mediated by PP4 is not sufficient to alter Smo subcellular localization. Interestingly, Cos2 is required for PP4 modulation on Smo [83]. As the direct interaction between PP4 and Cos2 was found, Cos2 may serve as a scaffold to associate PP4 and Smo, allowing the inhibition of Smo phosphorylation by PP4. Later, the involvement of PP4 in Hh pathway was reconfirmed in an in vivo screen [73]. However, these studies did not exclude a possibility that PP4 directly functions on Cos2, therefore indirectly modulate Smo phosphorylation. Further investigation for the *bona fide* target of PP4 in Hh pathway is expected.

### TAP42/ALPHA4

Alpha4 (Tap42 in yeast) is an atypical regulatory subunit, forming a complex with the catalytic subunit of PP2A, PP4, or protein phosphatase 6 (PP6). These three phosphatases are evolutionarily related, and together composing a protein serine/threonine phosphatase type 2A family [96–98]. The interaction between Alpha4 and each phosphatase catalytic subunit is independent of their canonical scaffolding and regulatory subunits [96, 97]. Alpha4 plays an important role in regulating the assembly and maintenance of PP2A phosphatase complexes, and its deletion leads to progressive loss of all PP2A, PP4 and PP6 phosphatase complexes [99]. RNAi-mediated silencing of *alpha4* altered the expressions of Hh signal related factors in *Drosophila* wing imaginal discs. The *alpha4* RNAi-induced effects were resulted from the loss of regulation of PP2A family members, as enforced expression of wild type *alpha4*, but not a phosphatase binding defective *alpha4* mutant, rescued the defective wing phenotypes [100], suggesting an essential role of Alpha4-regulated PP2A family phosphatase in Hh signal and wing development.

### Wild-type P53-induced phosphatase 1

Wild-type p53-induced phosphatase 1 (WIP1 or PPM1D) is a nuclear serine/threonine phosphatase expressed at low levels in most normal tissues [101]. In recent years, WIP1 has emerged as an important player in tumorigenesis [102]. The initial link between WIP1 and Hh signaling was established from a tumorigenesis study [103], in which ectopic expression of WIP1 enhances tumor formation in a Shh-dependent mouse model of medulloblastoma, one of most common tumors caused by improper Hh activity. A later study further elucidated the possible mechanism of WIP1 involving in Hh signaling [104]. Besides p53 as the known WIP1 target, Gli1 was also subject to the regulation from this phosphatase. WIP1 positively modulates Hh signaling by enhancing Gli1 transcriptional activity, nuclear localization, and protein stability. This modulation of Gli1 depends on WIP1 phosphatase activity and is p53-independent. It still remains mysterious whether WIP1 dephosphorylates Gli1 directly or indirectly through a third party.

### Lipid phosphatase

In addition to proteins, lipids are also subject to the regulation by phosphatase. Lipids, such as phosphoinositols, are major constitutes of plasma membrane and cellular organelle membrane, such as ciliary membrane. Given the importance of membranes in either *Drosophila* Hh pathway or vertebrate ciliary Hh pathway, it is worthy to note recent studies about the requirement of lipid phosphatases for normal Hh signal transduction [105, 106]. Primary cilium is a unique organelle for vertebrate Hh signal interpretation. Ciliary membrane contains a particular phosphoinositide, PI(4)P, whereas a different phosphoinositide, PI(4,5)P_2_, is located at the membrane of the ciliary base [106]. The level of PI(4,5)P_2_ at ciliary membrane is restricted by Inpp5e, a ciliary phosphoinositide 5-phosphatase, who selectively removes the phosphate from position d-5 of the inositol ring of phosphoinositides and inositol phosphates [107, 108]. In the *inpp5e*-deficient cilium, PI(4,5)P_2_ level is elevated and Hh signaling is disrupted [106]. In addition to defining lipid distribution, Inpp5e limits the ciliary localization of a PI(4,5)P_2_-binding protein, Tubby-like protein 3 (Tulp3), and its interacting proteins, intraflagellar transport complex A (IFT-A) and G-protein-coupled receptor Gpr161, all of which are negative regulators of Hh signaling [106, 109–113]. In *Drosophila*, although most cells are lacking cilium structure, PI(4)P is also critical for Hh signal transduction [105]. Hh-induced Smo release from Ptc inhibition and subsequent activation are dependent on the levels of PI(4)P. Correspondingly, another lipid phosphatase Suppressor of actin-1 (Sac1), which dephosphorylate PI(4)P, genetically functions downstream of Ptc in the regulation of Smo membrane localization and Hh pathway activation. Loss of Sac1 phosphatase results in *hh* gain-of-function phenotypes [105]. Together, different from the protein phosphatases mentioned above, lipid phosphatases, such as Inpp5e and Sac1, generate a specialized environment by controlling the protein/lipid composition at ciliary or plasma membrane, to facilitate Hh signal transduction.

## Phosphatase and HH morphogenetic response

**Fig. 3:**
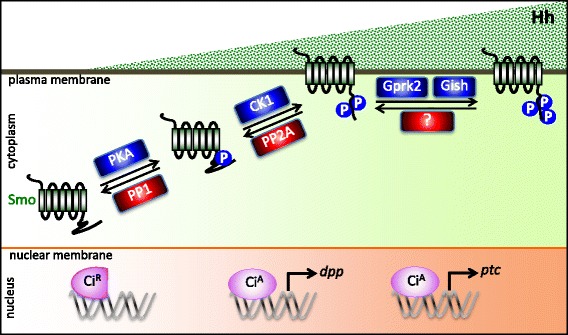
Multiple kinases-phosphatases mediated progressive Smo phosphorylations interpret morphogenic Hh signals in signal-receiving cells. In response to Hh concentration gradient, *Drosophila* Smo exhibits graded phosphorylation status, which correspondingly activates the expressions of a series of target genes, such as *dpp* and *ptc*, responding to low-to-intermediate threshold, or high threshold Hh signals, respectively. The transcription effector Ci is switched from a repressor form CiR to an activator form CiA, triggering the expression of Hh target genes. During these progressive Smo phosphorylations, PKA and CK1 sequentially phosphorylate Smo, which promotes Smo accumulation on plasma membrane and facilitates Smo further phosphorylations by Gprk2 and Gish, two kinases preferring the distribution near plasma membrane, to achieve maximal activation of Smo. It has been known that PP1 and PP2A respectively counteract with PKA and CK1 to modulate Smo phosphorylation status. However, the phosphatase against Gprk2 or Gish has not been identified yet

As a morphogen, Hh protein distributes over cells with a concentration gradient, which induces the different thresholds of signal cellular response in signal receiving cells, and eventually patterns the development of respective tissue or organs. During Hh signal transduction, phosphorylation has been implied to act as an important mechanism to not only fine-tune every component activity, but also interpret Hh morphogen gradient into graded downstream outcomes (Fig. 3).

The progressive Smo phosphorylations controlled by PKA-PP1 and CK1-PP2A have been illustrated to interpret morphogenic Hh signals into graded signaling outputs, which usually is represented by distinct thresholds of Hh-responsive gene expressions (Fig. 3) [72]. In responding to increasing Hh gradient, Smo obtains PKA-mediated intermediated level of phosphorylation and then CK1-regulated hyperphosphorylation [23]. The mutagenesis analyses have revealed that PKA-phosphorylated Smo species are sufficient to activate low-to-intermediate, but not high, threshold of Hh-responsive gene expressions, whereas PKA-primed CK1 phosphorylation is able to stabilize Smo at plasma membrane and induce the expression of high-threshold Hh target genes [23, 24, 72]. Correspondingly, by antagonizing kinase activities, PP1 or PP2A is capable of regulating the status of Smo phosphorylation and altering the expressions of Hh target genes [72]. Inhibiting the activities of all four PP1cs by nuclear inhibitor of protein phosphatase 1 (Nipp1), an endogenous inhibitor of PP1, is able to enrich PKA-phosphorylated Smo species, and induce the expressions of Ci and *dpp*, which are responding to low-to-intermediate Hh signals. Repression of PP2A activity is able to enhance Smo hyperphosphorylation by CK1 and activate the expression of *ptc*, a high-threshold Hh target gene.

In Hh pathway, Smo does not physically interact with either ligand Hh or receptor Ptc, therefore, the mechanism of how Smo obtains an order from Hh to undergo phosphorylation is not clearly characterized. Yavari et al. have proposed a model that Hh-Ptc binding alters the levels of PI4P at cell membrane to in turn regulate Smo plasma membrane localization and activation [105]. Even though, the relationship between membrane lipids and Smo phosphorylation is still elusive. Alternatively, it is possible that Hh regulates Smo phosphorylations through altering the activities of Smo-related kinases or phosphatases. Actually, upon Hh stimulation, the activities of PKA or CK1 were not obviously changed [72], making it likely that the regulation of PP1 or PP2A activities by Hh could be a major mechanism for Hh-induced Smo phosphorylation. It was observed a long time ago that okadaic acid-sensitive-phosphatase activity is induced by Shh treatment in cultured mammalian cells [80]. However, no investigation followed up to further dissect this observation and its underlying molecular mechanism. It will be of interest to delineate the Hh regulation in the expressions or activities of these related phosphatases in future. Regardless, the phosphatase study in Hh signaling has provided a new insight to fully understand the mechanisms of how the morphogenic Hh signals are transduced in cells.

## Challenges and opportunities in phosphatase study

**Fig. 4:**
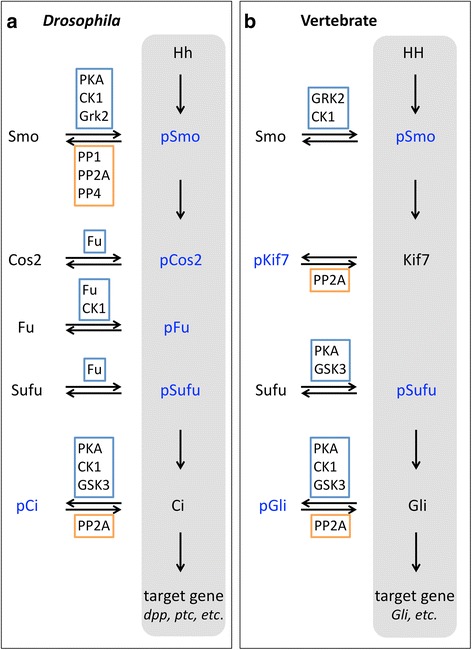
Summary of major kinases and phosphatases involved in Hh pathway. **a** In *Drosophila*, Hh induces the phosphorylations of Smo, Cos2, Fu, and Sufu proteins, but dephosphorylation of protein Ci, which activate the expressions of target genes. **b** Similarly, vertebrate Smo and Sufu proteins undergo phosphorylations, and Gli proteins undergo dephosphorylation upon HH stimulation. However, different from Cos2 in *Drosophila*, Kif7 is phosphorylated when HH signal is off, but dephosphorylated when HH signal is on. The major key kinases and phosphatases controlling these phosphorylation events are shown within blue boxes or orange boxes, respectively. Phosphorylated proteins are highlighted in blue. The signal transduction cascade under Hh condition is indicated with gray background

Although the current phosphatase study in Hh pathway has achieved remarkable progress (Fig. 4 a and b), it still falls far behind the kinase study. Up to now, the phosphatases affecting Cos2, Fu, or Sufu, remain mysterious. A few phosphatases have been identified to regulate Smo, Ci/Gli, and Kif7. However, the molecular basis of these phosphatase actions, including the specific targeting phosphor-residues on substrates, is largely unclear. The less progress on phosphatase study mainly is resulted from the difficulties apparently existing in this field.

First, it is a big challenge to correlate a phosphatase with its substrates if without any clues from experimental studies. Different from kinases that recognize their substrates simply depending on certain specific consensus sequence, a suitable binding of a phosphatase with its substrate relies on many aspects of these two proteins, such as their three dimension structures, conformations, and even the charges of residues. So it is nearly impossible currently to predict a substrate for a particular phosphatase, or search a responsible phosphatase for a known phosphor-protein, solely based on protein sequences. Second, many phosphatases execute functions only by forming complexes with one or more regulatory subunits. The function study for this type of phosphatase complex requires more comprehensive analysis for all components. Due to the high diversity of regulatory subunits, especially in vertebrates, a phosphatase may obtain various functions by binding with distinct regulatory subunits. But on the other side, the existence of multiple regulatory subunits also increases the complexity to delineate the function of a particular phosphatase, such as the occurrence of functional redundancy between different regulatory subunits.

To overcome these obstacles, many attempts have been undertaken. For example, much effort has been made to define a simple principle for the substrate recognition of PP1 or PP2A. A PP1-docking motif with well-defined consensus sequence RVxF was found to exist in about 70% of all PP1-interacting proteins including PP1 substrates [114]. For PP2A substrate recognition, a conserved LxxIxE motif was reported recently to provide a binding specificity to a particular PP2A phosphatase complex containing B56 regulatory subunit [115]. Although these motifs cannot fully represent the mechanisms to explain the substrate selection of PP1 or PP2A, it is a good starting point to search for PP1 or PP2A substrates. In addition, to bypass these sequence analysis, an organ-based genetic screen with a suitable readout is becoming a reliable way to search for the involved phosphatase under certain circumstances. For instances, several novel phosphatase regulators of Hh signaling, such as PPV and PpD3, have been identified in a screen through observing the expression pattern of Hh-responsive genes in *Drosophila* larval wing imaginal discs [73]. However, due to the way of phosphatase functioning as a complex and functional redundancy between different regulatory subunits or isoforms, it is expected that some phosphatase effectors could be missed from this kind of screens. Alternatively, according to the character of a protein serine/threonine phosphatase physically interacting with its substrate, utilizing biochemistry methods to precipitate the interacting proteins with a particular phosphatase could be another feasible approach to search for substrates of phosphatases [116]. With the improving techniques in proteomics and phosphatomics, such as phosphor-protein enrichment and advanced tandem mass spectrometry, identifying substrates for phosphatases through these biochemistry approaches appears to be more achievable now than before.

## Conclusions

During Hh signal transduction cascade, a broader phosphorylation spectrum has been outlined. As one of two key executors in phosphorylation process, the phosphatase has been increasingly studied in Hh pathway, and remarkable progress has been achieved in recent years. Many phosphatases have been identified in regulating Hh signal activities. Even though, the phosphatase study is still far away from the edge of completion. Many of known phosphorylation events in Hh pathway are lacking information of the responsible phosphatase. On the other side, the molecular mechanism by which the identified phosphatase regulators affect Hh signaling has not been clearly characterized. Fortunately, with the increasing emphasis and improving techniques for phosphatase studies, a more thorough understanding of the phosphatase functions in Hh pathway is promising in the near future.

## Abbreviations

aPKC: atypical protein kinase C
Cdc2l1: cell division cycle 2 like protein kinase 1
Ci: Cubitus interruptus
CiA: Ci activator form
CiR: Ci repressor form
CK1: casein kinase 1
CK2: casein kinase 2
Cos2: Costal2
DYRK: dual-specificity tyrosine phosphorylation-regulated kinase
Dzip1: Daz interacting protein 1
Fu: Fused
Gish: Gilgamesh
Gli: Glioma-associated oncogene homologue protein
GPCR: G-protein-coupled receptor
GRK/Gprk: G-protein-coupled receptor kinase
GSK3: glycogen synthase kinase 3
Hh: Hedgehog
HIB: Hh-induced MATH and BTB domain protein
Kif7: kinesin superfamily member 7
MAP3K10: mitogen-activated protein kinase kinase kinase 10
MEF: mouse embryonic fibroblast
OA: okadaic acid
PKA: protein kinase A
PP1: protein phosphatase 1
PP2A: protein phosphatase 2A
PP4: protein phosphatase 4
PPV: protein phosphatase V
Ptc: Patched
RNAi: RNA interference
Ser: serine
SPOP: speckle-type BTB/POZ protein
STK36: serine/threonine protein kinase 36
Sufu: Suppressor of fused
Thr: threonine
Tws: twins
Ulk3: Unc-51 like kinase 3
Wdb: widerborst
WIP1: wild-type p53-induced phosphatase 1
Wrd: well-rounded.
